# Linking Molecular Mechanisms and Evolutionary Consequences of Resource Polyphenism

**DOI:** 10.3389/fnint.2022.805061

**Published:** 2022-02-08

**Authors:** Nicholas A. Levis, Erik J. Ragsdale

**Affiliations:** Department of Biology, Indiana University, Bloomington, IN, United States

**Keywords:** competition, developmental switch, flexible stem, genetic assimilation, phenotypic plasticity, polyphenism

## Abstract

Resource polyphenism—the occurrence of environmentally induced, discrete, and intraspecific morphs showing differential niche use—is taxonomically widespread and fundamental to the evolution of ecological function where it has arisen. Despite longstanding appreciation for the ecological and evolutionary significance of resource polyphenism, only recently have its proximate mechanisms begun to be uncovered. Polyphenism switches, especially those influencing and influenced by trophic interactions, offer a route to integrating proximate and ultimate causation in studies of plasticity, and its potential influence on evolution more generally. Here, we use the major events in generalized polyphenic development as a scaffold for linking the molecular mechanisms of polyphenic switching with potential evolutionary outcomes of polyphenism and for discussing challenges and opportunities at each step in this process. Not only does the study of resource polyphenism uncover interesting details of discrete plasticity, it also illuminates and informs general principles at the intersection of development, ecology, and evolution.

## Introduction

**Figure 1:**
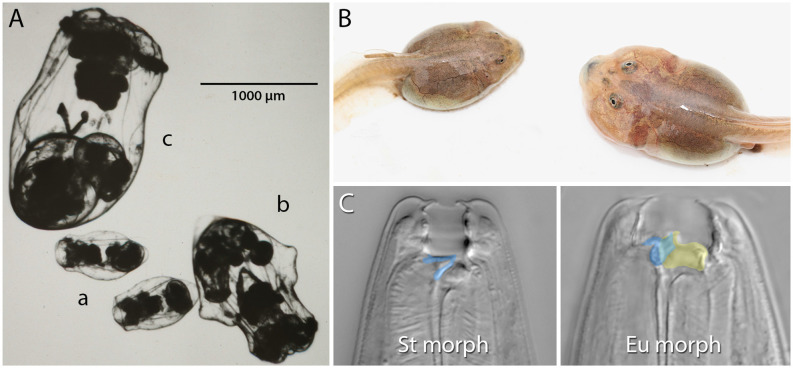
Examples of resource polyphenism. **(A)** Preserved saccate (a), cruciform (b), and campanulate (c) females of the rotifer *Asplanchna sieboldii*. These forms differ in their ability to capture and consume large prey items. **(B)** Omnivore (left) and carnivore (right) spadefoot toad (*Spea multiplicata*) tadpoles. In addition to the obvious size and jaw musculature differences, carnivores possess a shorter gut, more highly keratinzed mouthparts, and overall activity. Omnivore tadpoles primarily feed on detritus, but carnivores specialize on fairy shrimp and other tadpoles. **(C)** The stenostomatous (St morph) and eurystomatous (Eu morph) mouth forms of the nematode *Pristionchus pacificus*. False coloring indicates how the morphs differ in the shape of the dorsal tooth (blue) and the presence of an additional, opposing tooth (yellow). Whereas St worms are microbivores, Eu worms are omnivores capable of consuming both microbes (e.g., yeast, bacteria) and other nematodes. Image in **(A)** is courtesy of John J. Gilbert and reproduced from Gilbert (2017) with permission from John Wiley and Sons (© 2016 Cambridge Philosophical Society). Image in **(B)** is courtesy of David W. Pfennig and modified from Levis et al. (2020). Images in **(C)** are by Erik J. Ragsdale and from Bui et al. (2018).

**Figure 2:**
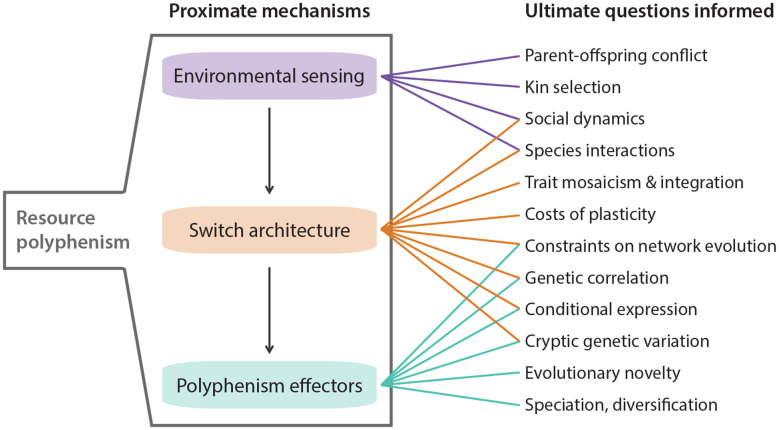
Highlighted areas where knowledge of resource polyphenism’s proximate mechanisms can inform ultimate questions about ecology and evolution.

Competition for resources is ubiquitous. Indeed, competition within and among species for limited resources formed the bedrock of Darwin’s arguments for the process of evolution by natural selection, and it has long been recognized for fostering diversification (Haldane, 1932; Van Valen, 1965; Roughgarden, 1972; Bolnick, 2001; Svanbäck and Bolnick, 2007; Maynard et al., 2017; Aristide and Morlon, 2019). In some cases, competitively mediated resource competition has led to the evolution of resource polyphenism (RP), or the occurrence within a single population or species of environmentally triggered alternative phenotypes showing differential use of niche or resources. In contrast to some resource-*dependent* polyphenisms (e.g., nutritionally based ones such as the development of large horns in well-fed male dung beetles; Moczek and Emlen, 1999), RP *per se* requires alternative resource *use*. RP has been documented across the tree of life (Figure 1) and includes ciliates (Ryals et al., 2002), rotifers (Gilbert, 2017), nematodes (Hirschmann, 1951; Kanzaki et al., 2019), insects (Pener and Simpson, 2009), fish (Nordeng, 1983), and amphibians (Pomeroy, 1981; Collins and Holomuzki, 1984). Despite this wide taxonomic representation, RP is not common. Nevertheless, RP nucleates diverse areas of biology, ranging from intra- and interspecific species interactions to molecular developmental mechanisms of phenotypic plasticity. Indeed, RP is unique among polyphenisms because its expression simultaneously is influenced by resource availability and influences other species that provide or compete for those resources. Thus, the study of RP acts as a nexus that informs diverse fields ranging from developmental genetics to community ecology (Figure 2).

The proximal mechanisms of RP are poorly understood in most organisms that have it. In general, polyphenic development requires individuals to assess their environment, especially in terms of available resources and/or the strength of competition for those resources, transduce environmental information to relevant signaling pathways and developmental switches, and ultimately adjust their developmental trajectories *via* modification of downstream regulatory networks (Nijhout, 2003; Ehrenreich and Pfennig, 2016; Projecto-Garcia et al., 2017; Lafuente and Beldade, 2019; Sommer, 2020). Each of these steps of polyphenic development has its own challenges and opportunities for studying the molecular, ecological, and evolutionary bases and consequences of RP. For example, proximal mechanisms of kin recognition, an important feature of RP, can help inform evolutionary questions like what drives differences in social interaction strategies and reproductive mode (Lightfoot et al., 2021; see section “Identification and Evolution of Environmental Sensing Mechanisms”). Determining the architecture of a developmental switch mechanism will enable predictions regarding the evolutionary outcomes of the phenotypes it regulates (see section “Evolution and Mechanisms of Developmental Switches”). Considering the molecular mechanisms of plasticity and RP also provides insights into the evolutionary constraints and how novel processes are integrated into existing ones (see section “Evolutionary Consequences of Resource Polyphenism Effectors”). As a more general example, by studying the molecular mechanisms of RP, especially in model organisms with abundant genetic tools, we can explore new dimensions of the nature and causes of ecologically impactful, developmental variation.

In this article, we use the major events in polyphenic development as a scaffold for linking molecular mechanisms with ultimate ecological and evolutionary outcomes and for discussing potential challenges and opportunities at each step in this process. Our goal throughout is to highlight recent developments and identify fruitful avenues for additional research.

## Identification and Evolution of Environmental Sensing Mechanisms

A universally acknowledged yet understudied aspect of phenotypic plasticity generally, and polyphenism specifically, is that organisms must reliably assess their environmental conditions. Such assessment is necessary because it allows the organism to determine the current conditions of the environment and, potentially, predict future conditions for itself and/or its offspring. Indeed, reliability of cues is often factored into models of plasticity (Lively, 1986; Tufto, 2000; Sultan and Spencer, 2002; Scheiner, 2013) and is known to be important for the evolution and maintenance of plasticity (Charnov and Bull, 1977; Moran, 1992; Bonamour et al., 2019). Despite their importance, relatively little empirical work has examined the mechanisms organisms use to assess their environment in order to shape the developmental responses of RP.

The limited studies that have explored environmental assessment in the context of RP have uncovered challenges unique to this form of plasticity. One of the most significant challenges is how to discriminate between kin and non-kin when both serve as competitors and potential food sources (Polis, 1981; Pfennig and Collins, 1993; Pfennig et al., 1994; Pfennig and Frankino, 1997; Pfennig, 1999). Kin recognition is particularly important for RP because many polyphenisms involve a predatory or cannibalistic form that would incur fitness costs if it were to consume closely related individuals. Even in non-cannibalistic cases, an individual would benefit from preferentially reducing competition with kin by adopting an alternative resource-use phenotype and using different resources than its relatives.

Mechanistically, how do resource-polyphenic taxa identify and subsequently avoid conflict with close relatives? From a holistic perspective, it is possible that multiple types of sensory information could inform an individual of its proximity to kin (Halpin, 1991; Tang-Martinez, 2001; Chung et al., 2020), but visual and auditory cues do not seem to be major modes of assessment in resource-polyphenic taxa in particular. Instead, tactile and chemical information appears to dominate, at least in those cases where an environmental assessment has been explored. For example, in tiger salamanders (*Ambystoma tigrinum*), larvae are more likely to develop into a cannibalistic morph if they are reared with non-kin (Pfennig and Collins, 1993); cannibal development requires tactile cues from other salamanders (Hoffman and Pfennig, 1999); and, once developed, cannibals discriminate between kin and non-kin using olfactory cues (Pfennig et al., 1994). This suggests that physical crowding and the scent of nearby individuals serve as major inputs controlling the decision to become cannibalistic. Likewise, in another amphibian with facultative cannibalistic larvae (*Spea multiplicata*), individuals are more likely to develop into cannibals when reared with non-kin (Pfennig and Frankino, 1997) and cannibals are better able to discriminate kin than non-cannibals are (Pfennig, 1999). In this case, kin identification might occur through tasting or “nipping” other tadpoles because, following a nip, non-siblings are more likely to be consumed than siblings (Pfennig et al., 1993). Kin recognition in this system may also be influenced by habitat selection and cues learned early in life regardless of source (Pfennig, 1990). In general, it seems that conspecific identity and kin recognition are important both for informing whether an individual develops into a carnivorous or cannibalistic form and for that form to navigate its social and competitive environment.

Recent studies in another animal group, resource-polyphenic nematodes of the genus *Pristionchus* (Figure 1C), have since revealed a proximal, molecular context for kin selection during predation. Not only do individuals of a species’ predatory morph kill individuals of other species at higher rates than they kill their own species, but even individual isolates (genotypes) of the same species kill other isolates at higher rates than their own (Lightfoot et al., 2019). Kin discrimination in this system largely depends on the identity of a hypervariable small peptide that is expressed in the nematodes’ body-wall epidermis (Lightfoot et al., 2019) and, otherwise, on overall genetic relatedness (Lightfoot et al., 2021). Although the cilia of anterior sensilla are required for prey assessment and environmental sensing in *P. pacificus*, self-recognition does not seem to require fully functioning cilia (Moreno et al., 2019), so it is not yet known how the peptide signal and genetic identity of others is detected.

For these nematodes, it is clear that conspecific identity matters once the predatory form develops, but it is unclear if the genetic identity of co-occurring conspecifics influences whether or not an individual develops into the predatory form. Preliminary evidence suggests that while the life-stage of co-occurring conspecifics influences the mouth-morph decision (with crowding cues from adult, but not juvenile, nematodes inducing the predatory morph), crowding cues from the same vs. a different strain did not differ in levels of induction (Werner et al., 2018). Yet, additional tests among diverse strains are needed to evaluate whether conspecific identity generally influences form induction. The above examples illustrate that kin recognition systems and the nature of kin selection are important ongoing areas of inquiry in resource-polyphenic taxa.

Another challenge facing the evolution of RP is how species gain the ability to reliably evaluate polyphenism-relevant environmental cues from existing sensory transduction pathways. Organisms depend on a restricted set of channels—specific stimuli, organs, cells, and molecular cascades—through which environmental information can be obtained, and these channels are likely used for more than guiding decisions about polyphenic development (e.g., predator avoidance). Therefore, this step in the development of a plastic trait might be relatively constrained in evolution. To highlight this point, we reconsider the nematode example above, in which it was not the sensory machinery that was evolving, but the signal (i.e., the hypervariable peptide) being sensed. Whether this expectation of evolutionary constraint is generalizable awaits additional empirical testing. One study addressing this issue, again in *P. pacificus*, found that phylogenetically conserved genes have been co-opted for temperature sensing as part of RP development (Lenuzzi et al., 2021). It is still unclear if this co-option involved the loss of ancestral functionality, a transition in functionality, or an addition to the functionality of these genes or their pathways. Answering this question will help inform how polyphenic developmental pathways evolve from monomorphic ones.

Going forward, identifying the proximal mechanisms through which resource-polyphenic taxa detect changes in their environment and the identity of conspecifics will help unravel the origins of polyphenism, specifically by inferring the lability and constraints by which existing sensory pathways are co-opted or integrated for novel responses. In addition, studying the social dynamics of resource-polyphenic taxa will continue to provide insights into the evolution of social interactions, kin recognition, kin selection, and possibly even altruism.

## Evolution and Mechanisms of Developmental Switches

Development can be described as a tree of branching switch points (Weismann, 1893; García-Bellido et al., 1979; Thomson, 1988; Raff, 1996; Peter and Davidson, 2017) able to take on a variety of higher-order structures (Lewis et al., 1977; Gardner et al., 2000; West-Eberhard, 2003, pp. 129–135; Erwin and Davidson, 2009; Davidson, 2010; Lugagne et al., 2017). Considering development as a series or network of switches accounts for semi-independence of an individual’s traits, continuous variation in modular traits, and variable condition sensitivity of trait expression and use, i.e., plasticity (West-Eberhard, 2003, ch. 5). Because they comprise the predictable, stepwise conduit of environmental information to phenotype, developmental switches should be where most evolution of plasticity *per se* occurs. Switches are the integration points for genetic and environmental information. Therefore, modifications to switches can adjust the conditional sensitivity of trait regulation, thereby altering its expression frequency and ultimately its exposure to selection. While this switch-based view of development generally holds for any phenotype, it is particularly important for polyphenisms. Here we discuss: some of the general ways in which developmental switches can be organized; what is known about some of the molecular details surrounding developmental switches in RP; and how various switch organizations might influence the evolution of RP.

As noted above, switch-based development can assume a number of organizations. We will now briefly describe some of these organizations and their potential consequences for evolution. First, we consider organization *via* “ganged switches” wherein a complex phenotype is produced by a sustained environmental signal that “turns on” a temporal series of subordinate switches (Mather, 1955; West-Eberhard, 2003, pp. 131–132). Ganged switches might involve a signal intensity component such that subsequent switches are activated by stronger and stronger cue intensity (sometimes called “serial switches”; West-Eberhard, 2003, pp. 132–133). With this type of organization, subordinate switches have different thresholds of responsiveness to the inducing cue, and premature inactivation or insufficient intensity of the cue results in the failure to develop the complex phenotype. A possible example of this switch architecture comes from *Asplanchna* spp. rotifers (Figure 1A). In *A. sieboldii* and related species, dietary acquisition of α-tocopherol from prey begins the multigenerational transition from a smaller saccate to a larger cruciform morphology (reviewed in Gilbert, 2017). The degree of body-wall outgrowth in the cruciform morph depends on the dose of α-tocopherol with reversion to only saccate individuals if α-tocopherol is removed. If α-tocopherol remains available and consumption of congeners and conspecifics occurs, depending on the species being studied, there is an additional transition from cruciform to the giant, campanulate form. Thus, the primary cue, α-tocopherol, needs to be sustained for full polyphenic conversion, and conversion takes place in a serial manner from saccate to cruciform to campanulate. A similar multigenerational RP transition has been described in the development of the gregarious phase of the migratory locust, *Locusta migratoria* (West-Eberhard, 2003; p. 132, and references therein).

Signal cascades consisting of a master switch (or “regulator”) that leads to an automatic deployment of one or another developmental pathway are another type of switch-based development (Gehring, 1998; Erwin and Davidson, 2009; Chan and Kyba, 2013; Davis and Rebay, 2017). In the case of a plastic response, passing a single threshold results in the development of the trait without downstream effectors being influenced by the environmental signal. A possible example of such a cascade occurs in the nematode *P. pacificus*. In this species, environmental cues determine the proportions of two key enzymes whose relative doses at a critical point in development leads to the activation of alternative gene regulatory networks and production of alternative, irreversible resource-use phenotypes in the adult stage (Bui and Ragsdale, 2019; Casasa et al., 2021). Inactivation of either enzyme results in the complete developmental conversion into one morph or the other (Ragsdale et al., 2013b; Bui et al., 2018; Namdeo et al., 2018). This enzyme-mediated decision is made through the nuclear receptor NHR-40, which is expressed in polyphenic tissue (Kieninger et al., 2016). Because NHR-40 is apparently the most downstream transcription factor known to control the switch itself, in contrast to the local morphologies the switch influences, we suppose that NHR-40 initiates subordinate cascades necessary to carry out the polyphenism decision. However, we still qualify this as a possible example because it is yet unclear if the activity of either enzyme or NHR-40 is needed to sustain development once a given morph’s regulatory network is activated or, alternatively, if the network only requires one-time activation with morph development following automatically. Disentangling a one-time cascade from the ganged-switch model requires close dissection of the chain reaction from environmental induction to phenotype expression.

Another example of a signal cascade mechanism regulating RP has been described for the ciliate *Tetrahymena vorax*. In this organism, a signal cascade is initiated by a prey-derived low-molecular weight metallocomplex binding to a putative cell surface receptor and facilitates the transition from the microstomal to the macrostomal form (Ryals et al., 2002). Because this switch characterizes feeding in organisms without development, it offers a chance to identify what general principles might unify or fundamentally distinguish proximal mechanisms in multicellular from single-celled RP. Nonetheless, signal cascades emanating from a master regulator can be a powerful way to elicit a phenotypic change without the need for a sustained environmental signal, as with ganged switches.

In addition to the temporally structured organizations described above, switches can be governed by spatial relationships and interactions, given the necessity of morphogen diffusion, cell-to-cell communication, and tissue-to-tissue communication for morphogenesis. In general, the activity of one phenotypic subunit (e.g., the growth or spread of that subunit) acts as a signal to adjacent subunits and thereby modifies their activity or development. Of course, these spatial interactions can also have a temporal component that utilizes concepts from the above mechanisms, such as sensitive periods of responsiveness and thresholds of responsiveness, to influence the efficacy and outcome of spatial switches. A possible example of the spatial influence on switches is the dorsal gland cell of *P. pacificus*. This cell was recently found to express a nuclear receptor (NHR-1) and several ultimate targets of that receptor that affect the mouth-dimorphism phenotype in that species (Sieriebriennikov et al., 2020). The functions of the identified polyphenism targets are still uncertain, leaving this example speculative. However, it is unlikely that the gland alone is required for the entire execution of the polyphenism switch because several other epithelial and myoepithelial cells of the nematode’s pharynx and surrounding tissue are also involved in producing the dimorphic morphology (Harry et al., 2021). We speculate that spatial interactions through connectivity to the dorsal gland may influence whether and how the polyphenism decision, once made, is fully realized. Indeed, NHR-1 mutants produce intermediate polyphenism phenotypes, suggesting that the receptor is necessary for the switch but insufficient for completely throwing it.

The final type of organization we mention has been referred to as “dispersed local switches”, “self-organization” (*sensu* West-Eberhard, 2003), or “developmental selection” (*sensu* Snell-Rood, 2012). This organization is similar to ganged switches in that there are several switches responding to the same environmental cue to produce a phenotype. However, in this case, the subunits are not serially activated but instead respond to locally experienced conditions. While not a case of RP, studies on seasonal color polyphenism of some butterfly wings have illustrated this type of switch (Rountree and Nijhout, 1995; Koch et al., 1996; Monteiro et al., 2015; van der Burg and Reed, 2021). Among examples of RP, such a switch structure might be responsible for development into the carnivore morph of spadefoot toad tadpoles (Figure 1B). Evidence for this possibility comes from observations that carnivores, which are initially induced by competition for and consumption of freshwater shrimp and other tadpoles, can revert into omnivores if environmental conditions change, such as a dramatic reduction in the availability of prey (Pomeroy, 1981; Pfennig, 1992a, b), as might happen following a second rain event at a pond (Levis et al., 2020). Often, these reverted individuals exhibit a mosaic of carnivore and omnivore features such as the short gut that is characteristic of carnivores and the jaw musculature of omnivores. These observations suggest that although the multiple component traits of the carnivore morph respond to the same environmental cues, different tissues respond to changes in the cue in different ways. Consistent with this idea, there is evidence suggesting that traits differ in the rate at which they transition between morphs (Pfennig, 1992b). Thus, the loss of cues or insufficient levels or duration of cues can result in “intermediate” and mosaic phenotypes. However, a recent study suggests that cholesterol biosynthesis and peroxisome activity might be important regulators of the morph transition because of their system-wide effects on metabolism and hormone production, suggesting a role for a top-down, “master regulator” model (Levis et al., 2021). In addition, whether the component traits of the carnivore morph are activated sequentially—thus making the architecture actually more akin to that of ganged switches—and are responding to the same internal (e.g., hormonal) signals, as opposed to environmental cues, requires further study.

Taking a step back, what can a switch-based view of development tell us about evolution, especially the evolution of polyphenism? Although this question has been addressed in detail (West-Eberhard, 2003), we focus instead on three aspects that we think are important in light of recent discoveries (see also Figure 2). First, depending on the specific architecture of the developmental switch, exposure to differences in duration and intensity of the environmental cue might have a dramatic impact on phenotype expressivity. For example, complete development of a complex trait under a ganged-switch model requires serial activation of switches by the same cue, and if that cue dissipates before the full phenotype is formed, then this incomplete development could open the door for natural selection by increasing the amount of variation in the form and function among the incomplete phenotypes. Alternatively, loss of the cue could lead to extinction if a deleterious, less-than-fully-formed phenotype results. Second, by considering switch architecture, we can also begin to predict which evolutionary outcomes might be expected following an environmental change (Snell-Rood et al., 2018; Levis and Pfennig, 2020). Dispersed local switches, for example, have the benefit of creating a high phenotype-environment match which might favor their evolutionary maintenance. However, if the environment becomes more stable, costs associated with this type of switch, such as increased time and energy spent sampling the environment, might make the evolutionary loss of polyphenism likely, as it might then favor genetic assimilation (reviewed in Snell-Rood, 2012). Third, switch architecture can help inform the degree to which subunits of a complex trait are free to evolve independently, their level of integration, and degree of modularity. For example, phenotypes produced by ganged switches might be less well-integrated than phenotypes depending on a master regulator (signal cascades) because the former requires a series of relatively independent activations, whereas the latter depends only on one. This is related to the first point above in that premature inactivation of the signal could decouple selection on the components of a ganged-switch phenotype, but is less likely to do so in a signal-cascade phenotype. In sum, considering the specific architecture of the switches involved in RP can provide powerful insights toward understanding the evolution of development in ecologically relevant contexts (Figure 2).

## Evolutionary Consequences of Resource Polyphenism Effectors

The evolutionary consequences following the origins of RP have received relatively thorough attention (e.g., Skúlason and Smith, 1995; Smith and Skúlason, 1996; Pfennig et al., 2010; and references therein). For example, the intraspecific variation and greater niche width wrought by RP have been suggested to decrease a lineage’s likelihood of extinction (Bradshaw, 1965). Likewise, the presence of RP might make competing species more likely to co-exist and prevent competitive exclusion (Orlando et al., 2011; Pfennig and Pfennig, 2012). RP has also long been considered an initial step toward speciation because some of the same factors involved in RP—spatial and/or temporal separation of alternative ecomorphs—can also favor assortative mating and reproductive isolation (Maynard Smith, 1966; Smith and Skúlason, 1996; West-Eberhard, 2003). In addition to simple lineage splitting, RP and plasticity more generally have also been implicated in driving adaptive radiation wherein various alternative resource use phenotypes to arise from a plastic ancestor (West-Eberhard, 2003). This model (sometimes referred to as the “flexible stem”) has increasing empirical support (Wund et al., 2008; Gibert, 2017; Schneider and Meyer, 2017). Thus, it is becoming increasingly compelling that RP contributes to diversification.

Speciation and lack of extinction are both ways by which RP might promote diversification. Indeed, a study by Pfennig and McGee (2010) found that taxonomic groups expressing RP had greater species richness than their sister groups lacking RP. More recently, a study by Susoy et al. (2015) found that, across 90 nematode species, the evolution of RP was associated with a subsequent increase in evolutionary rates and morphological complexity. Interestingly, the secondary loss of plasticity and resulting fixation of a single morph (i.e., genetic assimilation), also correlated with a strong increase in evolutionary rates. This suggests that currently monomorphic taxa that exhibited polyphenism in their evolutionary history might be influenced by, and benefit from, that history long after it has passed. The extent to which such a secondarily lost polyphenism influences subsequent evolution, especially through defined molecular components, is a relatively unexplored research frontier.

Not only does RP increase macroevolutionary diversity, it also fosters greater levels of intraspecific diversity. For example, Ragsdale et al. (2013a) identified an additional, exaggerated eurystomatous mouth form (dubbed “megastomatous”) in the resource-polyphenic nematode species *P. triformis*. In a more extreme example, some *Pristionchus* species have been found to produce up to five alternative morphs that fill diverse ecological niches (Susoy et al., 2016). This pattern of switches begetting switches and diversity begetting diversity is not restricted to nematodes. In the spadefoot toad tadpole example (Figure 1B), there has been subsequent diversification with some sub-morphs of the carnivore phenotype potentially specializing on alternative resources such as heterospecific tadpoles (Levis et al., 2017).

Finally, a growing body of literature supports a role for RP, and plasticity more generally, in driving the evolution of novelty (Moczek et al., 2011; Sommer, 2020; Levis and Pfennig, 2021). Thus, it is well-established that RP can have a significant impact on the evolutionary process by affecting diversity, novelty, and adaptation. However, our understanding of the molecular targets during these evolutionary processes is still in its infancy.

Emerging evidence from studies of plasticity more generally, and not just RP, suggest that evolution of plastic traits might most often occur by targeting genes and networks downstream of a primary switch mechanism (i.e., effector genes, or “environmentally sensitive loci”; Via et al., 1995). For example, a single-nucleotide change in downstream effectors caused constitutive or nearly constitutive development of matricidal hatching in the nematode *Caenorhabditis elegans* (Vigne et al., 2021) and of a low permeability heterocyst in the cyanobacterium *Fischerella thermalis* (Miller et al., 2020). Likewise, using artificial selection and diverse tests of genomic architecture and accessibility, van der Burg et al. (2020) showed that the evolutionary loss of plasticity in butterfly seasonal color pattern occurred through downstream changes to trait-specific genes, potentially through *cis*-regulatory changes. These studies support the idea that downstream components of a developmental hierarchy should be more labile to evolutionary modification (Hahn and Kern, 2005) because they are presumably less constrained by pleiotropy. Further consistent with this notion, Casasa et al. (2021) found that alternatively activated gene networks associated with RP in diplogastrid nematodes are overrepresented by rapidly evolving gene families.

Despite the growing evidence that evolution of plasticity might generally feature changes in effector genes, this issue is far from settled. We recall that the evolution of environmental sensing mechanisms (i.e., parts of the plastic response *upstream* of a developmental switch) in *P. pacificus* contributed to variation in temperature-dependent mouth form development (Lenuzzi et al., 2021). Moreover, the process of “building up” a polyphenism from a non-polyphenic ancestor likely requires more than a simple change to downstream effectors, as has been indicated by changes in regulatory genes themselves (Sieriebriennikov et al., 2018; Bhardwaj et al., 2020; Biddle and Ragsdale, 2020). Thus, while the above evidence might suggest that effector genes be the *a priori* hypothesis for the evolutionary targets of plasticity or at least its evolutionary loss, more work is needed, especially regarding the origins and maintenance of polyphenism.

Several important frontiers are open for integrating the well-known ecological and evolutionary outcomes of RP with molecular mechanisms. For instance, more empirical studies on the molecular mechanisms of conditional expression (*sensu* Van Dyken and Wade, 2010) are needed for us to better understand the extent to which alternative morphs of a polyphenism can evolve independently (Snell-Rood et al., 2010, 2011) and to predict which evolutionary outcomes might be most likely (Snell-Rood et al., 2018; Levis and Pfennig, 2020). Other important, unanswered questions include, if polyphenism facilitates speciation, do the loci involved in reproductive isolation overlap with those controlling polyphenic development? How are existing developmental networks rewired to incorporate novel information associated with polyphenism and associated novel traits? What is the nature of genetic variation that can be accumulated and released with the conditional expression of RP? Can we detect or infer a history of RP by investigating the gene regulatory network of an extant monomorphic species? Only by evaluating multiple, ideally replicate, instances of plasticity’s evolution, either in nature or in the lab, can we begin to unravel whether it follows general rules and patterns or whether it is idiosyncratic by the system.

## Conclusions

We have described three major steps involved in generating a polyphenic developmental response, highlighted recent efforts to understand the mechanisms controlling each step, and alluded to some of the ongoing challenges and opportunities that remain. We briefly revisit some of these challenges and opportunities here (see also Figure 2). First, we need additional work to understand how polyphenic taxa assess the status and kinship of conspecifics and how they integrate polyphenism-related sensory mechanisms with more general sensory machinery. Exploring these issues will inform more general principles such as kin selection, trait integration, co-option, and the evolution of novelty. Second, fine-scale studies on the molecular bases of developmental switching will help answer several questions regarding the generalizability of plasticity mechanisms. For example, are “master regulators” more common than dispersed local switches? How do additive vs. epistatic effects influence developmental switching (Goldstein and Ehrenreich, 2021)? How do particular genetic architectures affect what evolutionary outcomes are possible? Third, the ecological and evolutionary consequences of RP have been studied for decades. However, a developmental genetic understanding of these consequences is relatively lacking. By considering these mechanisms, we can get a better grasp on the costs of plasticity vs. costs of phenotypes and how they affect evolution (Murren et al., 2015), how trait regulation shifts between high and low environmental sensitivity, and the interplay between developmental capacitance and adaptive evolution (e.g., Moczek, 2007). In short, the time is ripe to dive deeper into the molecular mechanisms, switch architectures, and evolutionary consequences of RP. Doing so should have profound implications for understanding how plasticity influences the nature and cause of variability.

## Author Contributions

NL conceived the manuscript and wrote the initial draft. NL and ER contributed to the final development of the manuscript and its structure and content. Both authors contributed to the article and approved the submitted version.

## Conflict of Interest

The authors declare that the research was conducted in the absence of any commercial or financial relationships that could be construed as a potential conflict of interest.

## Publisher’s Note

All claims expressed in this article are solely those of the authors and do not necessarily represent those of their affiliated organizations, or those of the publisher, the editors and the reviewers. Any product that may be evaluated in this article, or claim that may be made by its manufacturer, is not guaranteed or endorsed by the publisher.
